# Methionine-mediated synthesis of magnetic nanoparticles and functionalization with gold quantum dots for theranostic applications

**DOI:** 10.3762/bjnano.8.174

**Published:** 2017-08-22

**Authors:** Arūnas Jagminas, Agnė Mikalauskaitė, Vitalijus Karabanovas, Jūrate Vaičiūnienė

**Affiliations:** 1 State Research Institute Center for Physical Sciences and Technology, Sauletekio Ave. 3, LT- 10222, Vilnius, Lithuania; 2 National Cancer Institute, Baublio 3b, LT- 08406, Vilnius, Lithuania

**Keywords:** functionalization, gold, magnetic nanoparticles, quantum dots, theranostics

## Abstract

Biocompatible superparamagnetic iron oxide nanoparticles (NPs) through smart chemical functionalization of their surface with fluorescent species, therapeutic proteins, antibiotics, and aptamers offer remarkable potential for diagnosis and therapy of disease sites at their initial stage of growth. Such NPs can be obtained by the creation of proper linkers between magnetic NP and fluorescent or drug probes. One of these linkers is gold, because it is chemically stable, nontoxic and capable to link various biomolecules. In this study, we present a way for a simple and reliable decoration the surface of magnetic NPs with gold quantum dots (QDs) containing more than 13.5% of Au^+^. Emphasis is put on the synthesis of magnetic NPs by co-precipitation using the amino acid methionine as NP growth-stabilizing agent capable to later reduce and attach gold species. The surface of these NPs can be further conjugated with targeting and chemotherapy agents, such as cancer stem cell-related antibodies and the anticancer drug doxorubicin, for early detection and improved treatment. In order to verify our findings, high-resolution transmission electron microscopy (HRTEM), atomic force microscopy (AFM), FTIR spectroscopy, inductively coupled plasma mass spectroscopy (ICP-MS), and X-ray photoelectron spectroscopy (XPS) of as-formed CoFe_2_O_4_ NPs before and after decoration with gold QDs were applied.

## Introduction

In current nanomedicine, biocompatible iron oxide-based NPs have attracted particular interest due to their size-dependent magnetic, optical and chemical properties that allow for the design of NPs for multimodal imaging and photothermal therapy of cancer cells [1]. Dual-imaging probes, capable to perform simultaneously magnetic resonance and fluorescent imaging, allow for a more rapid and precise screening of the oncological disease sites. This is frequently achieved by covering magnetic NPs with shells containing luminescent quantum dots (QDs) [2–6]. The target molecules can be attached to the surface of magnetic NPs through biocompatible links such as Au–S– [7]. Iron oxide NPs can be coated with polymeric or silica shells containing incorporated gold NPs [8–10]. However, in this case the size of the magnetic NPs increases up to ten times [9], resulting in a significant decrease in the saturation magnetization value of the magnetic core. To eliminate this drawback, several methods for the deposition of the gold directly onto the surface of magnetic NPs have been proposed that are based on the reduction of Au(III) species by the typical reducing agents such as borohydride, ascorbic acid and citric acid [11–14]. However, the direct-deposition protocols are mainly suitable for covering γ-Fe_2_O_3_ NPs. The formation of a gold shell on magnetite (Fe_3_O_4_) or ferrite surfaces through reduction of chloroauric acid by citrates or borohydride is usually problematic due to the formation of pure gold crystallites in the solution [5,15]. The deposition of gold onto the surface of magnetic iron oxide-based NPs can also be achieved via their impregnation with hydroxylamine [16], vitamin C [17] or methionine [18–19], which are capable to reduce the gold ions at the surface of NPs. However, in this case, uniform coating of magnetic NPs can only be obtained via precise control of the precursor content and all steps of the multistep process [17–18]. As a result, this way is time-consuming and it does not fully prevent the formation of gold crystallites in the plating solution. Moreover, to avoid the aggregation of magnetic NPs during or at the end of the synthesis they must be covered with capping materials such as acid anions [20–21], surfactants [22] or proteins [23]. Besides, for in vivo and in vitro applications of magnetic NPs their capping materials should be biocompatible and allow for the attachment of gold species. In recent publications amino acids such as methionine [19] and lysine [24] have been reported to be effective capping agents to control the size of magnetite [19] and Co ferrite [24] NPs during co-precipitation synthesis [25]. The main goal of the methionine capping was the application of Fe_3_O_4_@Met NPs for the adsorption of water pollutants.

In this study, we report a novel synthesis protocol for superparamagnetic cobalt ferrite NPs capped with a biocompatible methionine shell (CoFe_2_O_4_@Met), which in turn is capable to reduce and attach the gold species. In this way, hybrid magneto-plasmonic cobalt ferrite NPs decorated with Au^0^/Au^1+^ quantum dots (QDs) were formed for the first time. The formation of plasmonic gold QDs at the surface of iron oxide-based NPs was confirmed by HRTEM, AFM, FTIR, XPS and chemical analysis.

## Results and Discussion

### Synthesis and characterization of methionine-functionalized cobalt ferrite nanoparticles

**Figure 1:**
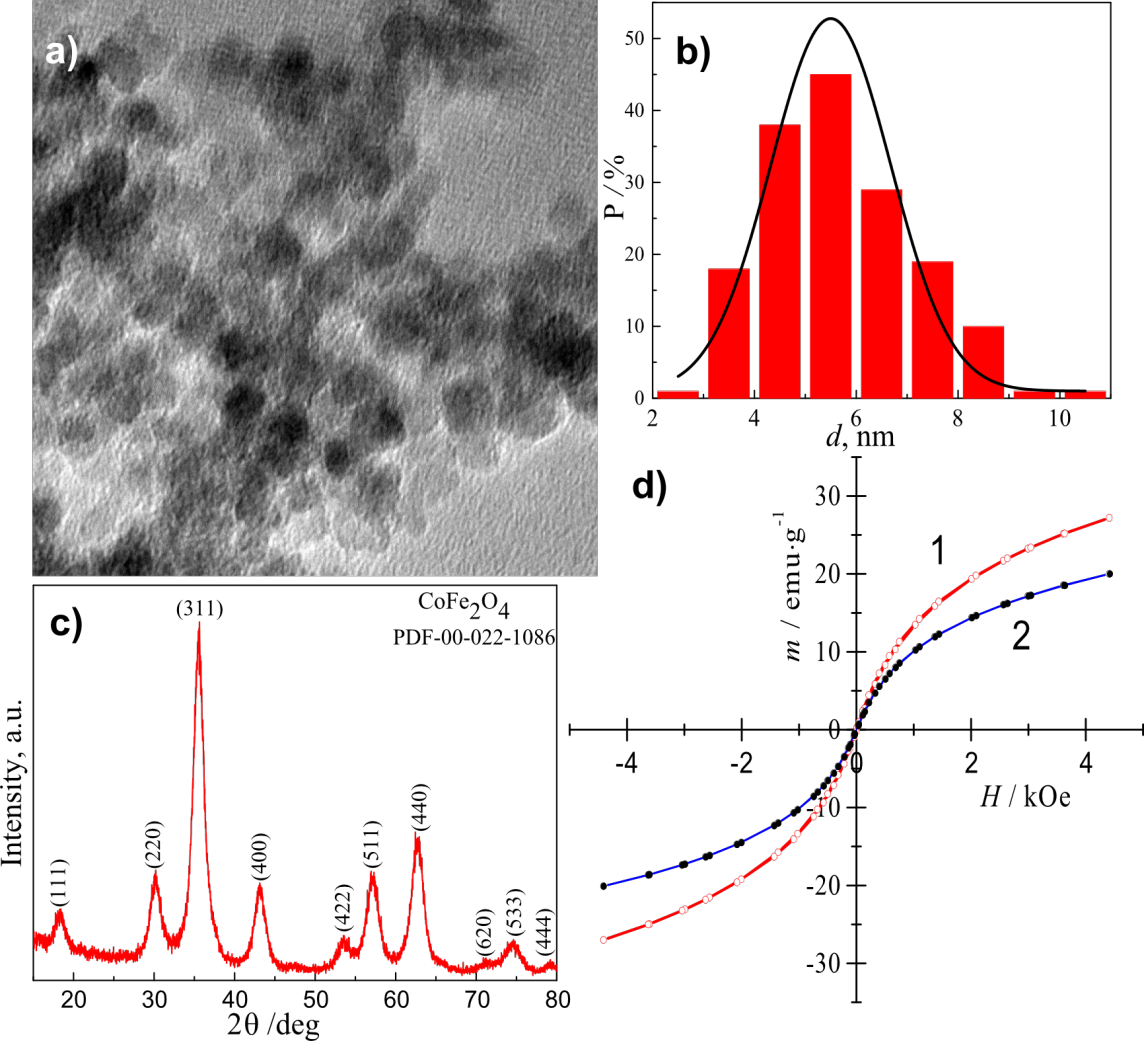
a) TEM image of cobalt ferrite NPs synthesized hydrothermally in a solution containing 25.0 mmol·L^−1^ CoCl_2_, 50 mmol·L^−1^ FeCl_3_, 0.2 mol·L^−1^ methionine, and NaOH to pH 12.4 at 130 °C for 10 h. The size distribution histogram and XRD pattern of the as-formed NPs are shown in panels b) and c), respectively. In panel d) the magnetic responses of as-formed (1) and sonicated NPs in a 10 mmol·L^−1^ HAuCl_4_ solution, kept at a pH 12.2, at 37 °C for 4 h (2) are presented.

A hydrothermal approach was applied to synthesize the superparamagnetic cobalt ferrite NPs stabilized with methionine. The proposed approach differs from the reported one [19] in the nature of magnetic NPs, the composition of the aqueous solution applied, synthesis atmosphere and modes. It involves the preparation of an alkaline aqueous solution containing CoCl_2_, FeCl_3_, methionine, and NaOH up to pH 12.4, followed by autoclaving at 130 °C for 10 h. To the best of our knowledge, methionine has not been applied before for hydrothermal synthesis and stabilization of cobalt ferrite NPs as the capping ligand and reducing agent of gold ions. The interest in NPs capped with methionine was based on the current understanding that methionine can reduce chloroauric acid from alkaline solutions anchoring Au^0^ at the surface of the NPs [18]. As-synthesized NPs were characterized by TEM, XRD, FTIR and magnetic measurements. Figure 1a depicts the TEM image of the as-grown NPs that have been carefully rinsed and reveals their spherical shape and a size distribution in the range of (3.0 – 8.5) nm with a mean value of 5.7 nm (Figure 1b). Furthermore, the stabilization of cobalt ferrite NPs with metionine molecules confers them strong non-fouling properties not allowing aggregate. The XRD pattern of these NPs (Figure 1c) implied the formation of pure, inverse spinel structure CoFe_2_O_4_, as all diffraction peaks at 2*Θ* positions: 18.29 (111), 30.08 (220), 35.44 (311), 43.06 (400), 53.45 (422), 56.97 (511) 62.59 (440), and 74.01 (533) match well with the standard polycrystalline CoFe_2_O_4_ diffraction data summarized in the PDF Card No. 00.022-1086. The average size of as-grown Nps, calculated by the Scherrer formula [26] from the (311) XRD line broadening ~ 6.0 nm, it is a close proximity to the one calculated from the TEM data (5.8 nm, Figure 1b).

**Figure 2:**
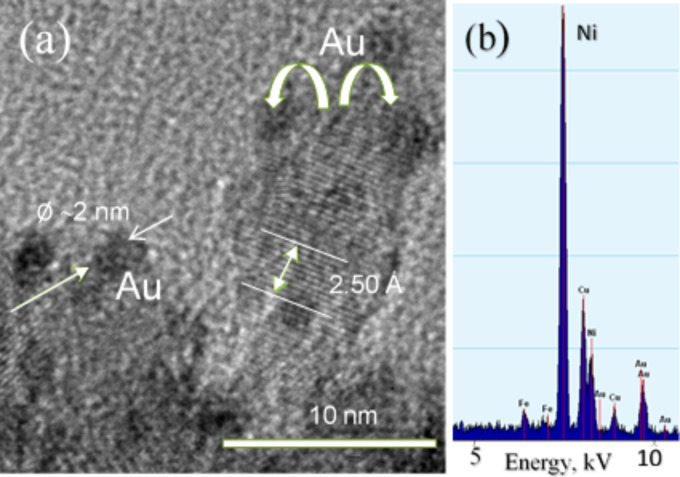
HRTEM image of CoFe_2_O_4_@Met NPs after sonication in 15 mmol·L^−1^ HAuCl_4_ solution at 37 °C for 4 h (a) and their EDX spectrum (b).

Magnetization measurements were further performed to evaluate the gold deposition onto the surface of cobalt ferrite NPs. Figure 1d shows the room-temperature magnetization plots as a function of applied magnetic field for CoFe_2_O_4_@Met NPs before (1) and after (2) their sonication in the chloroauric acid solution. It was found that the saturation magnetization value of CoFe_2_O_4_@Met NPs decreases from 27 to 21 emu·g^−1^ (at *H*_max_ = 4.4 kOe) upon sonication supporting the claim that gold species are deposited but the NPs remain superparamagnetic. The high-resolution TEM image of the CoFe_2_O_4_@Met NPs after gold deposition with methionine and the EDX spectrum of these NPs are shown in Figure 2.

**Figure 3:**
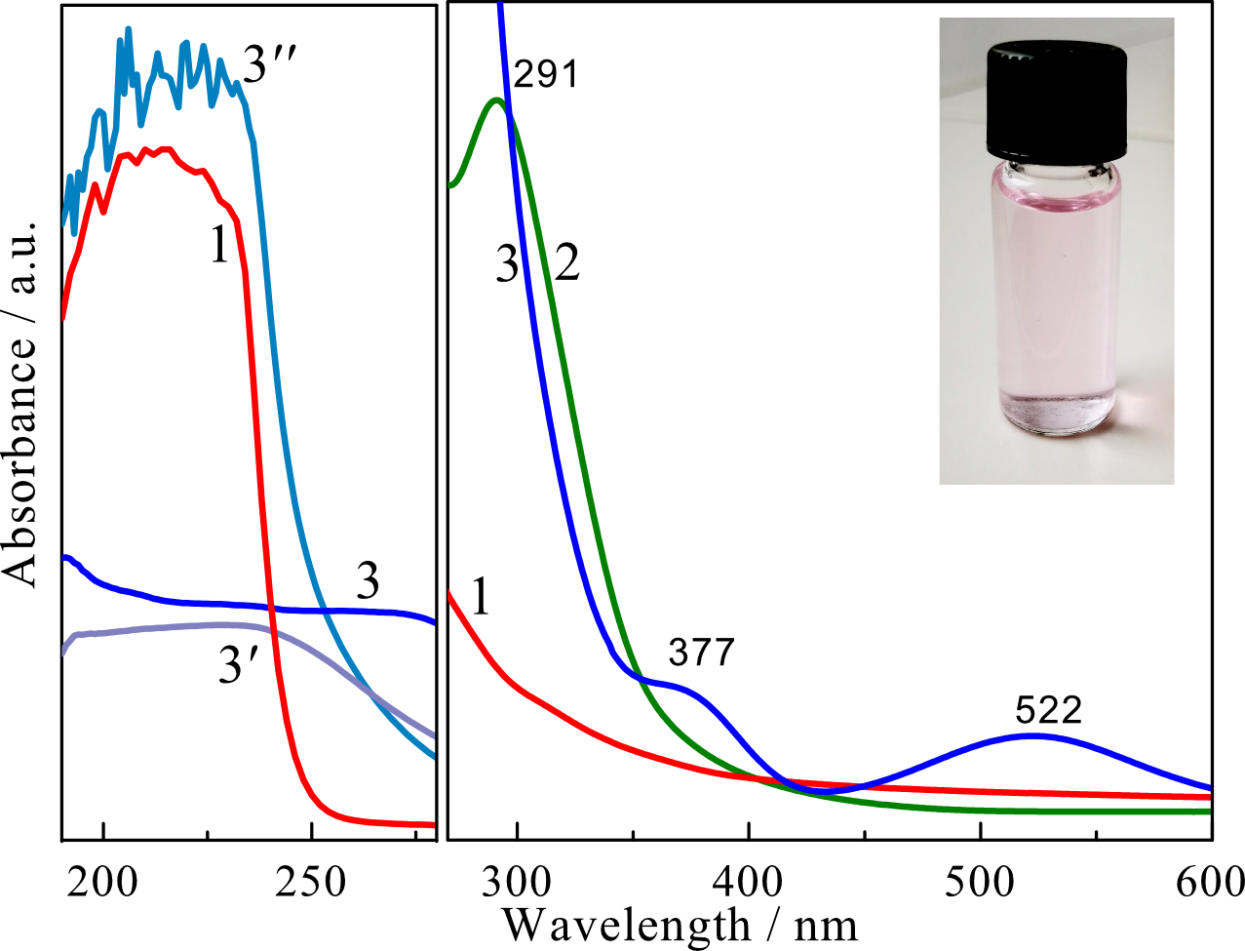
Absorption spectra of methionine (1), tetrachlorauric acid (2) and reddish-pink colored solution of gold species (inset) collected from the CoFe_2_O_4_@Met–Au nanoparticles initially (3) and after dilution to one half (3′) and to on quarter (3″).

The HRTEM image shows the formation of numerous gold species at the surface of methionine-stabilized CoFe_2_O_4_@Met NPs. In accordance with HRTEM image and EDX spectrum, the ICP-MS analysis of the gold plating solution performed before and after 30 min of sonication of the NPs indicated the reduction of ca. 99.3% of gold ions. From the HRTEM inspection, however, it was difficult to determine the size distribution of the attached gold species, although some of them seemed to be spherical with a diameter of ca. 2.0 nm. More precise results were obtained by the determination of the size of gold species that were removed from the NP surface by the ultrasonic agitation of 10 mg CoFe_2_O_4_@Met/Au NPs probe in 10 mmol·L^−1^ methionine solution. As a result a reddish-pink solution was obtained after 20 min processing (see inset in Figure 3). This process is most likely due to the stronger capping of Au NPs with methionine molecules than with CoFe_2_O_4_@Met/Au NPs. Note that no fluorescence was seen under UV and blue-light excitation of this solution. Typical UV–vis absorption spectra of aqueous methionine, tetrachlorauric acid and gold species solution are shown in Figure 3.

**Figure 4:**
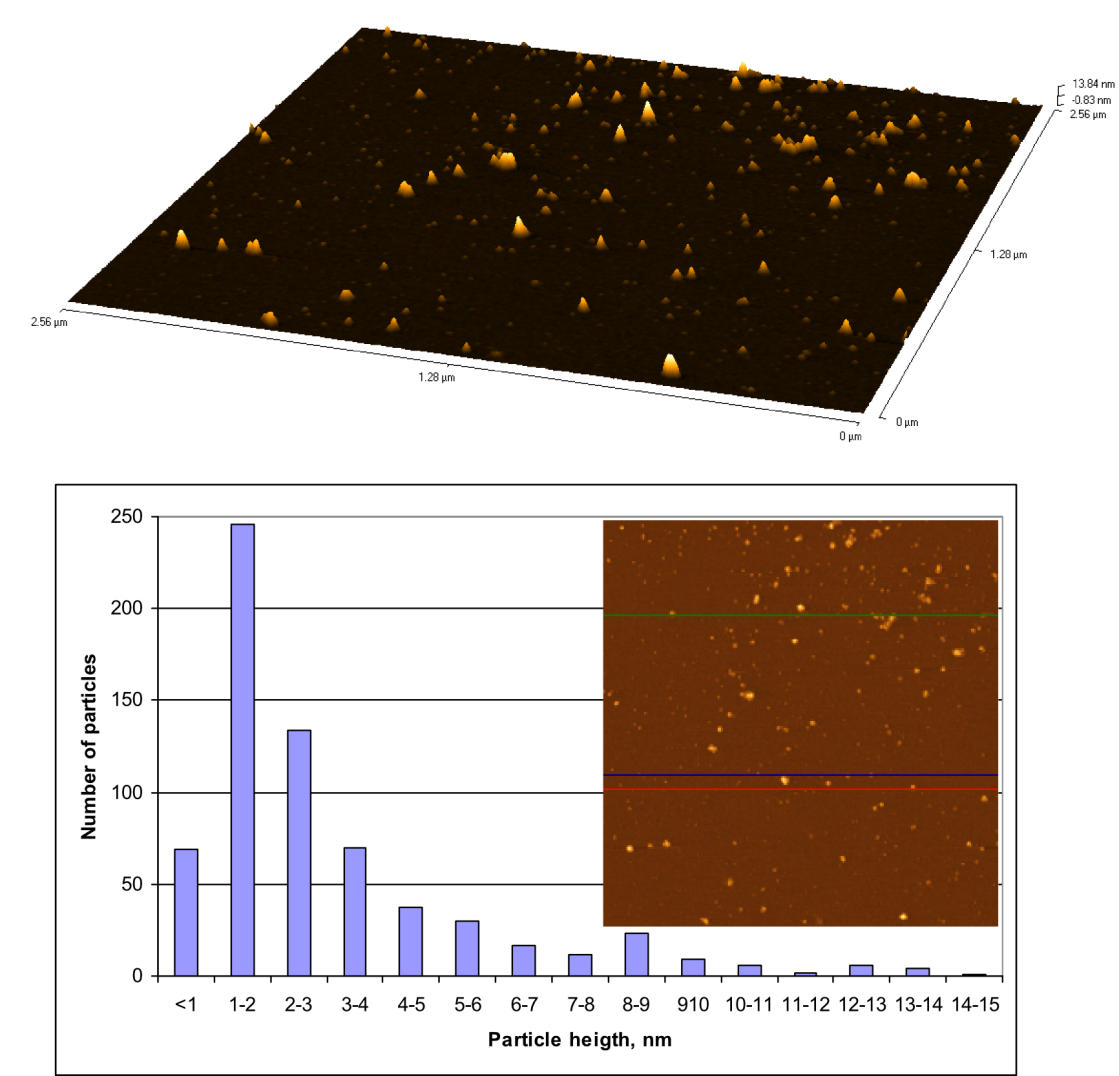
a) AFM 3D image and b) size distribution histogram b) of Au species removed from the surface of CoFe_2_O_4_@Met–Au NPs.

The pure methionine solution does not exhibit any absorption peaks in the measured spectral range. For the chloroauric acid solution, however, a clearly resolved absorption peak at 291 nm is observed. The UV–vis absorption spectrum of the solution containing the gold species collected from the surface of the cobalt ferrite NPs (Figure 3, plot 3) exhibits two absorption shoulders at 522 and 377 nm. The former seems to be originated from the surface plasmon absorption of metallic Au [27–29]. The position of this band mainly depends on the size of Au species [30]. So the absorption position of this peak indicates that the size of the methionine-stabilized gold species is extremely small. This assumption was further verified by AFM of gold species spread on a freshly cleaved mica substrate (Figure 4a).

According to these investigations, the shape and size of gold species attached to the surface of magnetic NPs were estimated. The vast majority of species are 1–2 nm sized gold quantum dots (QDs) (Figure 4b). Control experiments demonstrated that the gold species detached from the surface of magnetic NPs coalesced upon dilution of the analyzed Au@Met solution. Consequently, it can be assumed that a significant part of the NPs larger than 2–3 nm are coalesced ultra-small gold QDs.

**Figure 5:**
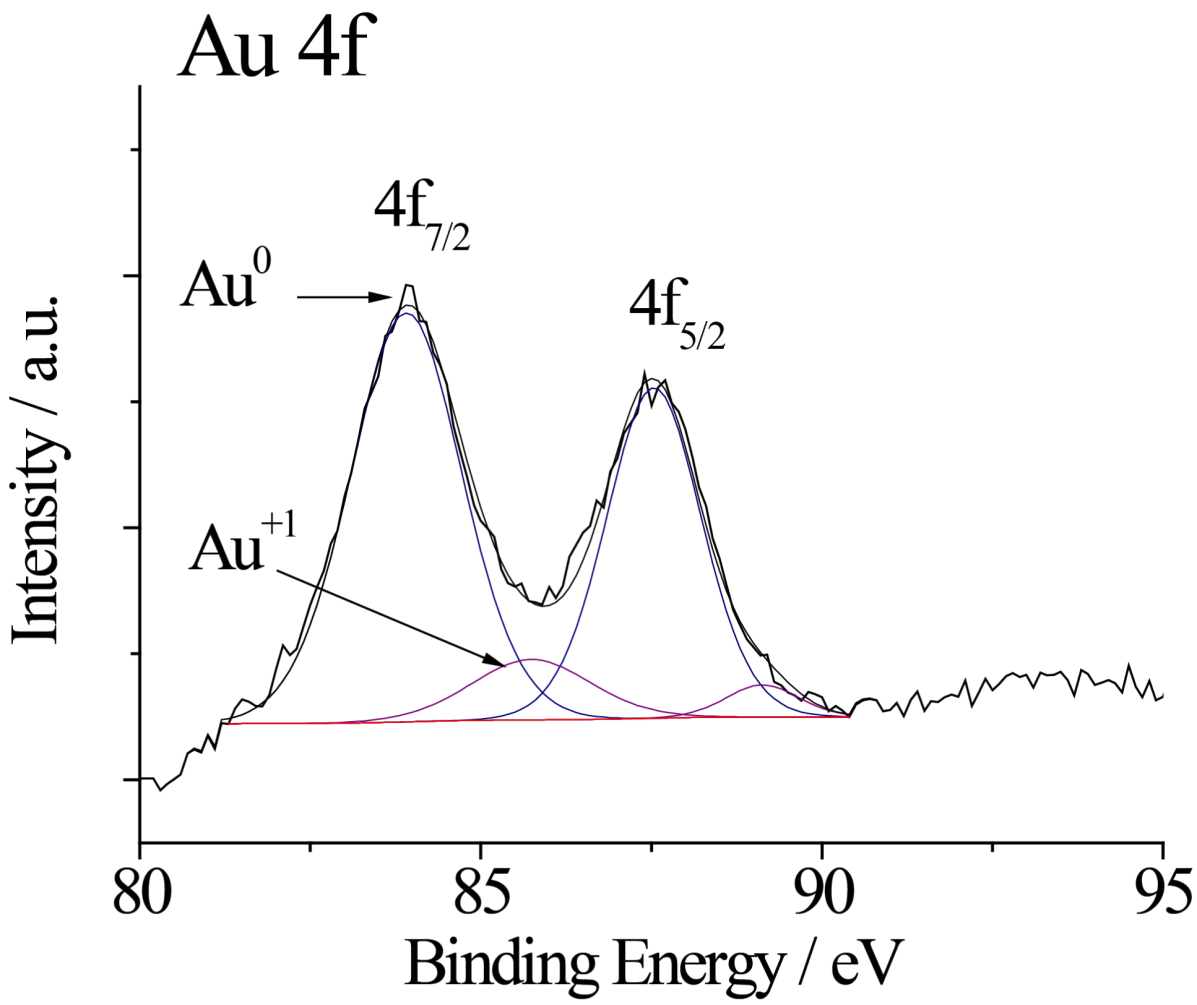
Deconvoluted X-ray photoelectron spectrum (XPS) of Au 4f.

The state of gold species formed and attached to the surface of methionine-stabilized cobalt ferrite NPs was also investigated using X-ray photoelectron spectroscopy (XPS). The surface chemical composition of the CoFe_2_O_4_@Met–Au NPs is presented in Table 1, whereas the typical core-level spectrum of the deposited gold is presented in Figure 5. As shown, the main Au 4f_7/2_ photoelectron peak is located at a binding energy (BE) value of 83.94 eV, typical of pure metallic Au^0^ species [31]. The fitting of the Au 4f core-level spectrum is performed further by using two spin–orbit split Au 4f_7/2_ and Au 4f_5/2_ components, separated by 3.56 eV. Surprisingly, the Au 4f curve fitting shows an additional shoulder peaked at 85.74 eV indicating the presence of Au^+^ species [31–32]. Their relative distribution reveals a fraction of about 13.7% of Au^+^ on the NPs surface of the total deposited gold content of 1.39% (Table 1). It is noticeable that plasmonic gold NPs upon excitation with nanosecond laser light the wavelength of which corresponds to the maximum absorption peak can create hot electrons in the conductive band of gold and, as a result, generate especially active singlet oxygen (^1^O_2_), **^·^**OH and O_2_^−^ [33–34].

**Table 1:** Elemental composition of CoFe_2_O_4_@Met–Au NPs.

name	peak BE (eV)	FWHM (eV)	peak area (arb. un.)	atom %

Au 4f	83.94	1.96	12435.07	1.39
C 1s	284.87	2.88	18041.56	36.02
N 1s	399.98	2.24	2647.25	3.02
O 1s	530.21	3.03	55974.26	40.37
Fe 2p	710.75	3.70	63210.72	12.68
Co 2p	780.67	3.29	36815.35	6.47

### FTIR spectra

**Figure 6:**
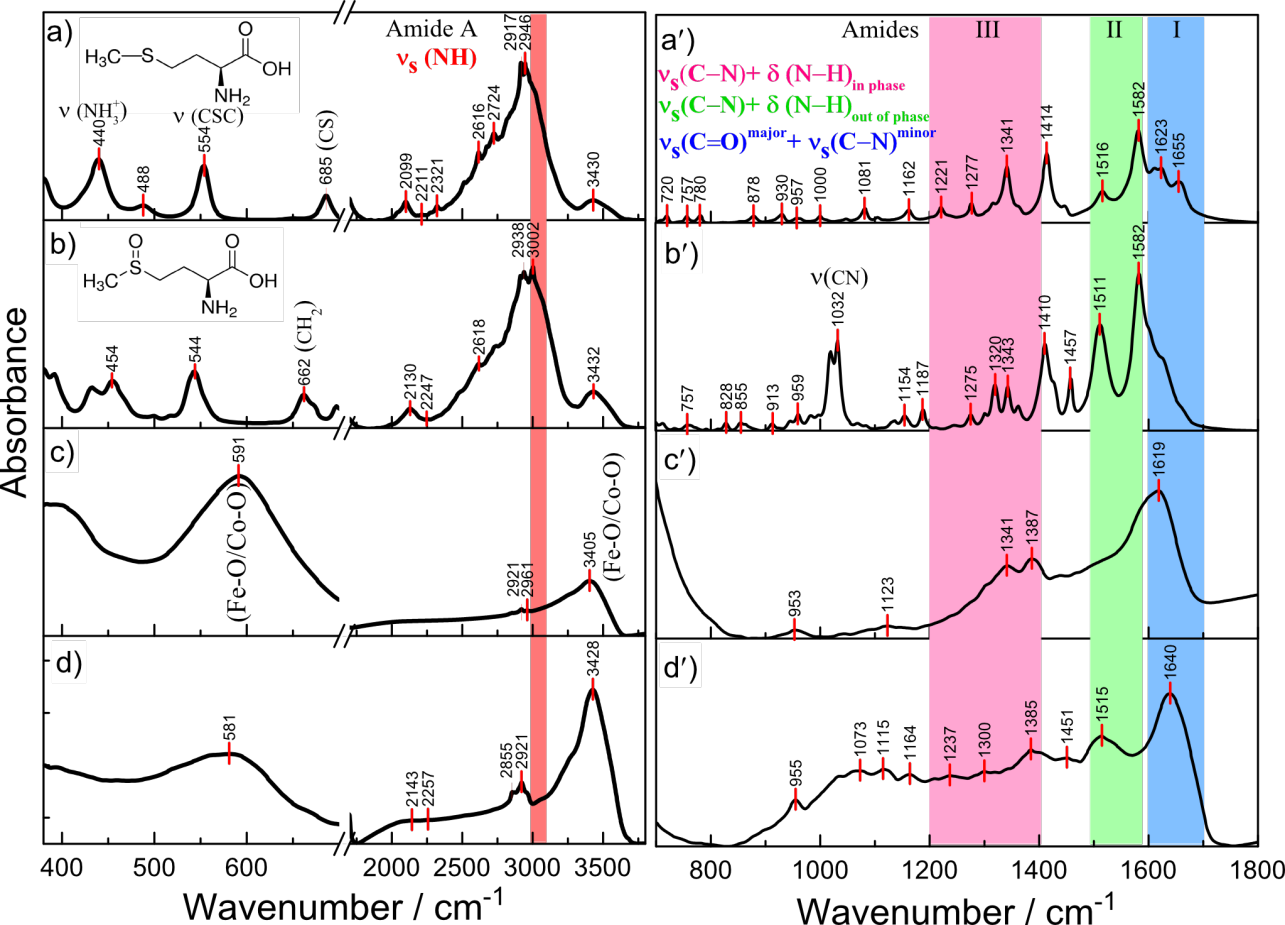
FTIR spectra of methionine (a, a′), methionine sulfoxide (b, b′), cobalt ferrite NPs stabilized with methionine (c, c′), and the same NPs after decoration with gold (d, d′) within the indicated wavenumbers.

Figure 6 compares the infrared spectra of cobalt ferrite NPs grown via the methionine-assisted hydrothermal approach, and methionine as well as methionine sulfoxide. The FTIR spectrum of the same NPs sonicated in an aqueous solution of chloroauric acid at 37 °C for 4 h is presented. The characteristic peaks of methionine are at 1582 cm^−1^, assigned to antisymmetric ν_as_(COO) and symmetric ν_s_(COO) stretching vibrations of the COO^−^ group, whereas the bands in the spectral region of 1277–1341 cm^−1^ are due to the coupled vibration of CH_2_ antisymmetric deformation and CH deformation modes [35–36]. According to the literature data [27], the band at 1516 cm^−1^ is associated with the symmetric deformation vibration of NH_3_^+^, δ_s_(NH_3_). Besides, the typical methionine S–C stretching mode at 685 cm^−1^ [37–38] and a clear resolved C–S–C stretching mode, ν(CSC), peaked at 554 cm^−1^ [39] are present in the spectrum. In the FTIR spectra of methionine and methionine sulfoxide a broad and strong band peaked at 2950–3002 cm^−1^ belongs to the symmetric stretching of NH_3_^+^ ions [40]. In the spectrum of Co ferrite NPs, presented in Figure 6c, the intense and broad band peaked at 591 cm^−1^ belongs to Fe–O/Co–O stretching vibrations in the tetrahedral metal complex [41]. The broad band, peaked near 1515 cm^−1^, belongs to δ_s_(NH_3_) mode and is indicative of the presence of charged amino groups [35,37]. The symmetric C–H deformation mode is also observed at 1341 cm^−1^ in the FTIR spectra of both pure methionine and CoFe_2_O_4_@Met. The attachment of methionine molecules during the synthesis of NPs can also be proven by the presence of the vibration modes in the frequency range of 2961–2855 cm^−1^, attributable to the symmetric stretching of NH_3_^+^ ions [42]. The frequency of ν_s_(COO) downshifts from 1414 to 1387 cm^−1^ upon stabilization of ferrite NPs with methionine molecules. The band near 1515 cm^−1^, however, can only be seen in the CoFe_2_O_4_@Met FTIR spectrum after sonication of NPs in the chloroauric acid-containing solution. The well-resolved band peaked at 1385 cm^−1^ is also characteristic for the FTIR spectrum of NPs after their sonication in the chloroauric acid solution (Figure 6d). As has been previously reported, such frequency downshift is due to the direct interaction of the carboxylate group of the amino acid with the NP surface [43]. We also suspect that the appearance of the significantly stronger symmetric vibration mode in the FTIR spectrum of gold decorated NPs at 1515 cm^−1^ due to cooperative vibrations of –CH_3_ and –NH_2_ groups is indicative of the oxidation of methionine to methionine sulfoxide. However, this mechanism requires more specific evidence and needs to be studied.

## Conclusion

Superparamagnetic methionine-coated cobalt ferrite nanoparticles with an average size of ca. 6 nm were hydrothermally synthesized via co-precipitation. Then the stabilizing shell of methionine molecules attached to Np surface was successfully applied for the reduction of the chloroauric acid. The formation of ultra-small Au^0^/Au^+^ QDs with a mean size of ca. 1.5 nm at the surface of magnetic NPs, which retains their magnetic, binding and conjugation properties, has been confirmed by HRTEM, AFM, XPS and magnetic investigations. Contrary to the previous works reported on the formation of Au^0^ nanoparticulate shells with thicknesses above 10 nm, we obtained numerous Au^0^/Au^+^ QDs at the surface of magnetic NPs stabilized with a biocompatible methionine shell. In this way, the initial saturation magnetization of the CoFe_2_O_4_@Met NPs (ca. 27 emu·g^−1^) decreased by ca. 22%. Besides, the formation of more than 13.5% of extremely active Au^+^ species of the total gold content at the surface can have a dramatic effect on the formation of the surface protein corona in the bloodstream that affects CoFe_2_O_4_@Met–Au NPs passive targeting and uptake into tumor cells.

The elaborated functionalization of magnetic NPs with gold QDs represents a promising multi-task platform for linking magnetic NPs with specific targeting ligands, such as aptamers and antibodies. This synthesis way may also be explored in future to design superparamagnetic, methionine-stabilized plasmonic magnetite NPs decorated with Au^0^/Au^+1^ QDs.

## Experimental

**Chemicals:** All chemicals, including Co(II) and Fe(III) chlorides, and HAuCl_4_·4H_2_O were of analytical grade, purchased from Aldrich and used without further purification. NaOH was purchased from Poch SA (Poland) and purified by preparation of a saturated solution, which lead to crystallization of other sodium salts. D,L-methionine (99% purity) and D,L-methionine sulfoxide (≥99.0% purity) were purchased from Sigma-Aldrich Co. Distilled water was used throughout the experiments.

**Synthesis of Co-ferrite nanoparticles:** Superparamagnetic cobalt ferrite nanoparticles were synthesized by a hydrothermal approach in an alkaline solution (40 mL) of Co(II) and Fe(III) chlorides, at a molar ratio 1:2, at 130 °C for 10 h using a 10 K·min^−1^ ramp. The total metal salt concentration was 75 mmol·L^−1^. Methionine (0.2 mol·L^−1^) was used as the reducing and capping additive. The pH value of the solution was kept at 12.4 by addition of 2.0 mol·L^−1^ NaOH solution. The required quantity of NaOH solution was determined by an additional blank experiment. In the subsequent experiment, this quantity was placed in the reactor, and mixed with the other components, during several seconds under vigorous stirring. The as-grown products were collected by centrifugation at 8500 rpm for 3 min and carefully rinsed 5 times using fresh portions (10 mL) of H_2_O. Afterwards, the NPs were dried at 60 °C. The collected NPs were studied and subjected to further processing within the following two days.

**Gold deposition:** The deposition of gold onto the Co ferrite surface was carried out through the methionine-induced chemical reduction of HAuCl_4_. Briefly, 3.5 mL of NP solution was diluted to 5 mL under ultrasonic agitation for 10 min and 2.0 mL of HAuCl_4_ (10 mmol·L^−1^) was introduced into the reaction medium under ultrasound agitation. The solution was alkalized to the required pH value by addition of 2.0 mol·L^−1^ NaOH under vigorous stirring. The deposition process was performed at 37 °C for 4 h under mild mixing conditions. The products obtained were collected by magnetic separation, carefully rinsed several times with deionized water and re-dispersed in ethanol for further examinations. For TEM observations, a drop of NPs suspension was placed onto a lacey grid, whereas for FTIR and magnetic investigations the suspension was dried at 60 °C.

**Analysis:** The concentration of gold remaining in the deposition solution was determined by inductively coupled plasma mass spectrometry. Measurements were made on emission peaks at λ_Au_ = 267.595 nm, λ_Au_ = 242.795 nm, λ_Co_ = 228.616 nm and λ_Fe_ = 238.204 nm using an OPTIMA 7000DV (Perkin Elmer, USA) spectrometer. Calibration curves were made using dissolved standards (1 to 50 ppm) in the same acid matrix as the unknown samples.

**Characterization:** The morphology of as-grown products was investigated using a transmission electron microscope (TEM, model MORGAGNI 268) operated at an accelerating voltage of 72 keV. The average size of nanoparticles was estimated from at least 150 species observed in the TEM images. High-resolution transmission electron microscopy (HRTEM) studies of as-synthesized products were performed using a LIBRA 200 FE at an accelerating voltage of 200 keV. X-ray powder diffraction experiments were performed on a D8 diffractometer (Bruker AXS, Germany), equipped with a Göbel mirror as a primary beam monochromator for Cu Kα radiation. Upgraded vacuum generator (VG) ESCALAB MKII spectrometer, fitted with a new XR4 twin anode, was used for XPS investigations. The non-monochromatised Mg Kα X-ray source was operated at *h*ν = 1253.6 eV with 300 W power (20 mA/15 kV) and the pressure in the analysis chamber was lower than 5 × 10^−7^ Pa during spectral acquisition. The spectra were acquired with an electron analyzer pass energy of 20 eV and resolution of 0.05 eV and with a pass energy of 100 eV. All spectra were recorded at a 90° take-off angle and the binding energies (BE) scale was calibrated by measuring of the C 1s peak at 284.6 eV. The spectra calibration, processing and fitting routines were done using Avantage software (5.918) provided by Thermo VG Scientific. Core-level peaks of Fe 2p, Co 2p, Au 4f, C 1s and O 1s were analyzed using a nonlinear Shirley-type background and the calculation of the elemental composition was performed on the basis of Scofield’s relative sensitivity factors. The FTIR spectra were recorded in transmission mode with a Bruker Vertex 70v vacuum FTIR spectrometer over the wavenumber range of 4000–400 cm^−1^. A 7 mm thick KBr discs were prepared under high pressure by mixing the powdered samples with KBr powder. Samples for AFM measurements were prepared by casting a drop (20 µL) of gold NP solution on freshly cleaved V-1 grade muscovite mica (SPI supplies, USA). The drop of solution was removed after 60s by spinning the sample at 1000 rpm. The commercially available atomic force microscope (AFM) diInnova (Veeco instruments inc., USA) was used to take three-dimensional (3D) images of gold nanoparticles. TESPA-V2 cantilevers (Veeco Instruments Inc., USA) with a tip curvature of 8 nm were used. Measurements were performed in the tapping mode in air. Images were acquired at the scan rate of 1 Hz per line with the 512 × 512 pixel image resolution. Image processing included flattening (2nd order) to remove the background slope caused by the irregularities of the piezoelectric scanner. The analysis was performed using the SpmLabAnalysis software (Veeco Instruments Inc., USA).

Magnetization measurements were accomplished using a vibrating-sample magnetometer calibrated by a Ni sample of similar dimensions as the studied sample. The magnetometer was composed of the vibrator, the lock-in amplifier, and the electromagnet. The magnetic field was measured by a testameter FH 54 (Magnet-Physics Dr. Steingrover GmbH).
